# Clinician awareness of brain computer interfaces: a Canadian national survey

**DOI:** 10.1186/s12984-019-0624-7

**Published:** 2020-01-06

**Authors:** Sasha Letourneau, Ephrem Takele Zewdie, Zeanna Jadavji, John Andersen, Lee M. Burkholder, Adam Kirton

**Affiliations:** 1 Department of Pediatrics, Cumming School of Medicine, University of Calgary, 2500 University Drive N.W., Calgary, AB T2N 1N4 Canada; 2 Clinical Neurosciences, Cumming School of Medicine, University of Calgary, 2500 University Drive N.W, Calgary, AB AB T2N 1N4 Canada; 3 Department of Pediatrics, University of Alberta, 116 St. and 85 Ave, Edmonton, AB T6G 2R3 Canada; 4 Alberta Children’s Hospital Research Institute, 28 Oki Drive S.W, Calgary, AB T3B6A8 Canada; 5 Hotchkiss Brain Institute, University of Calgary, 2500 University Drive N.W, Calgary, AB T2N 1N4 Canada

**Keywords:** Brain computer interface, Stroke, Spinal cord injury, Rehabilitation, Quality of life, Neuro-rehabilitation, Cerebral palsy, Neuromuscular disorders

## Abstract

**Background:**

Individuals with severe neurological disabilities but preserved cognition, including children, are often precluded from connecting with their environments. Brain computer interfaces (BCI) are a potential solution where advancing technologies create new clinical opportunities. We evaluated clinician awareness as a modifiable barrier to progress and identified eligible populations.

**Methods:**

We executed a national, population-based, cross-sectional survey of physician specialists caring for persons with severe disability. An evidence- and experience-based survey had three themes: clinician BCI knowledge, eligible populations, and potential impact. A BCI knowledge index was created and scored. Canadian adult and pediatric neurologists, physiatrists and a subset of developmental pediatricians were contacted. Secure, web-based software administered the survey via email with online data collection.

**Results:**

Of 922 valid emails (664 neurologists, 253 physiatrists), 137 (15%) responded. One third estimated that ≥10% of their patients had severe neurological disability with cognitive capacity. BCI knowledge scores were low with > 40% identifying as less than “vaguely aware” and only 15% as “somewhat familiar” or better. Knowledge did not differ across specialties. Only 6 physicians (4%) had patients using BCI. Communication and wheelchair control rated highest for potentially improving quality of life. Most (81%) felt BCI had high potential to improve quality of life. Estimates suggested that > 13,000 Canadians (36 M population) might benefit from BCI technologies.

**Conclusions:**

Despite high potential and thousands of patients who might benefit, BCI awareness among clinicians caring for disabled persons is poor. Further, functional priorities for BCI applications may differ between medical professionals and potential BCI users, perhaps reflecting that clinicians possess a less accurate understanding of the desires and needs of potential end-users. Improving knowledge and engaging both clinicians and patients could facilitate BCI program development to improve patient outcomes.

## Background

Few circumstances are more tragic than an intellectually capable individual trapped inside a body that cannot move. Unfortunately, multiple pediatric and adult neurological conditions, including cerebral palsy (CP), amyotrophic lateral sclerosis (ALS), brainstem stroke, and spinal cord injury (SCI), can create such locked-in syndromes. Accurate prevalence rates for the number of persons severely affected by these conditions are lacking but estimated to be in the thousands in Canada (population ~ 36 million) [1]. Treatment options are limited with one consequence being that affected individuals are deprived of their fundamental human rights, including being able to interact with their world.

Brain computer interface (BCI) technologies have major potential to improve quality of life for such persons. BCI works by first detecting patterns in brain signals associated with specific mental activities, such as imagining movements or mental arithmetic. Features are then extracted from these signal patterns and fed through a computer-based, translational algorithm that converts the brain’s electrical activity into device commands. These commands may be used to control a variety of effector devices including a computer cursor, communication system, or robotic arm [2]. Invasive BCI systems require implantation of sensors directly into the brain and marked advances continue to occur with such systems [3–8]. Meanwhile, non-invasive BCI systems that typically employ surface electroencephalography (EEG) have also advanced over the last few decades [6, 8]. Much poorer signal-to-noise in non-invasive systems is countered by more practical clinical utility as compared to invasive systems [9, 10]. Simple, wireless, economical, dry, EEG-based non-invasive BCI systems continue to evolve and may be used to perform basic tasks, even by young children with minimal training [9–12]. In addition to potentially liberating patients with severe motor impairment, BCI applications are also increasing across other areas of neurorehabilitation such as stroke [5, 6, 13]. While invasive BCI may be less practical due to cost and the need for surgery, their performance still far exceeds current non-invasive BCI [10, 14, 15]. Thus, the continued co-evolution of both invasive and non-invasive systems promises new opportunities for severely disabled persons to realize greater independence.

Despite this remarkable potential, translation of BCI use into clinical patient populations has been slow. Possible reasons may include significant technological challenges in generating reliable and user-friendly non-invasive systems, high inter-individual variability in the neural signals used, fears and risks associated with implantation of more reliable invasive BCI systems and disease-related alterations in nervous system physiology [9, 10]. As progress in BCI development continues to surmount these challenges [6, 8, 16], additional clinical barriers must also be considered. Over 90% of BCI studies have been conducted on healthy individuals rather than patients. Also under-served are children affected by such conditions (e.g. quadriplegic cerebral palsy) who face decades of life living with severe morbidity. There is also a paucity of input toward BCI development from the clinicians who understand the neurobiology of the diseases and provide ongoing care for the patients and families affected. It is hoped that clinically practical BCI systems will become increasingly available in the coming years [7, 8, 17]. To realize this potential to impact larger numbers of affected patients, an evaluation of BCI awareness by relevant clinicians and characterization of eligible patient populations are required.

## Methods

We conducted a national, population-based survey study with two primary aims. First, we assessed specialist physicians’ knowledge of BCI technology and, in doing so, aimed to increase physician awareness of BCI. Second, we wanted to estimate the number of patients in Canada who may benefit from BCI technology. We hypothesized that specialist physicians’ BCI knowledge is poor despite thousands of eligible Canadians with severe disabilities who might benefit.

This was a prospective, cross-sectional, national, online, questionnaire survey study. A nationally regulated specialist board certification system provided opportunity for population-based sampling. The study was approved by the University of Calgary Conjoint Health Research Ethics Board.

### Survey design

An initial set of potential topics were created based on broad review of the BCI literature and clinical experience of the project team. These topics were transformed into narrative and Likert scale questions to generate a pilot survey. Practicing specialists with relevant clinical expertise, including a neurologist, a physical medicine and rehabilitation specialist (hereafter “physiatrist”), a developmental pediatrician and a biomedical engineer research scientist with expertise in BCI reviewed the survey and provided feedback which was incorporated into a final version (Additional file 1). The survey was put online using REDCap, a secure web-based survey software. Multiple mock trials of the online survey were completed for quality assurance. Collected data was stored on the secure REDCap server and de-identified data was exported to Microsoft Excel for analysis.

### Participants

A multi-step strategy was employed to optimize survey distribution to all eligible Canadian specialists in adult and pediatric neurology and physical medicine and rehabilitation, medical specialties estimated most likely to have a high degree of exposure to clinical populations who could benefit from BCI. First, names of eligible specialist physicians were acquired from the Royal College of Physicians and Surgeons of Canada (RCPSC) Directory [18]. Second, websites of all Canadian medical schools (*n* = 16) as well as major hospitals (*n*~ 40) were screened to identify eligible physicians. Third, the lead investigators of two recent national surveys of Canadian adult and child neurologists [19, 20] provided names of relevant physicians. These names were cross-referenced to create a final list reviewed by at least one specialist member of the study team to screen for omissions. Surveys were disseminated to recipients via a public link sharable amongst colleagues with relevant practices.

Inclusion criteria were physicians currently practicing adult or child neurology, physiatry, developmental pediatrics or pediatrics. A small number of pediatricians and developmental pediatricians were included because they were grandfathered as pediatric neurologists or had pediatric neurology practices. For this reason and modest numbers, they were analyzed as part of the pediatric neurologist group. Physicians not able to complete the survey in English or not currently licensed or practicing in Canada were excluded. Participants were required to confirm they were currently licensed to practice their self-identified specialty.

From the final list generated above, email addresses were collected through multiple sources, including university and hospital websites, published journal articles, and Google searches. Invitation emails were sent (blind-copy) from the lead investigator (AK). The invitation email included a brief study overview (aim, participant involvement, incentive), terms of agreement for participation and provision of informed consent, and the link to the survey. The implied consent form was attached to the email. Contact information for the lead investigator and the research ethics board was provided. The first recruitment email was sent in June 2017 with a single reminder sent after 10 days. The survey was anonymous, contained no specific identifiable information, and responses were not linked to a participant ID. Upon survey completion, participants were redirected to an optional page which they could complete to enter a draw for a $300 Chapters/Indigo or Amazon gift card.

### Survey content

The survey had three primary components: 1) Demographics; 2) Baseline BCI knowledge and 3) Estimation of relevant clinical populations.

The “Demographics” section queried physicians’ specialty and subspecialty, experience, geography and catchment population. Physicians self-identified as adult or pediatric neurologists, physiatrists, developmental pediatricians or pediatricians. Lists of adult neurology, pediatric neurology and physiatry subspecialties were generated from the literature [19, 21]*.* Within each specialty, respective subspecialties were then classified *a priori* as “BCI-related” or “non-BCI-related” based on estimates of relative opportunity for exposure to BCI-eligible patients (Table 1).

**Table 1:** *A priori *dichotomization of subspecialties

Specialty	Subspecialties	
	“BCI-related”	“Non-BCI-related”
Adult Neurology	• Spinal cord injury • Stroke • Amyotrophic lateral sclerosis • Cerebral palsy • Critical care/emergency neurology • Neuromuscular disorders	• Alzheimer’s disease • Acquired brain injury/traumatic brain injury • Behavioural neurology • Brain tumour • Epilepsy • Headache/migraine • Movement disorders • Multiple sclerosis • Neuro-ophthalmology • Neuro-oncology • Pain/palliative • Sleep disorders
Pediatric Neurology	• Spinal cord injury • Stroke and perinatal stroke • Cerebral palsy • Critical care/emergency neurology • Neuromuscular disorders	• Acquired brain injury/traumatic brain injury • Behavioural neurology • Brain tumour • Epilepsy • Headache/migraine • Movement disorders • Multiple sclerosis • Neuro-ophthalmology • Neuro-oncology • Pain/palliative • Sleep disorders
Physiatry	• Spasticity management • Spinal cord injury • Stroke • Neuromuscular disorders • Prosthetics and orthotics	• Acquired brain injury/traumatic brain injury • Electrodiagnostic medicine • Geriatric rehabilitation • Musculoskeletal medicine • Paediatric rehabilitation • Pain management • Pulmonary, cardiac and cancer rehabilitation • Rheumatology

Legend:* A priori* dichotomization was based on each specialty’s estimated likelihood of leading physicians to interact with patients who could benefit from BCI.

In the “Baseline knowledge of BCI” section, participants rated their current knowledge level regarding 13 statements about BCI. These statements were carefully constructed to also provide a foundation of knowledge about BCI with which they could answer the rest of the survey questions. Baseline BCI knowledge responses were scored using the following Likert scale: 0 = “No Knowledge”; 1 = “Vaguely Aware”; 2 = “Somewhat familiar”; 3 = “Very familiar.” The mean of each physician’s responses to the 13 statements was calculated to create a “BCI knowledge score” which was used to compare physicians’ overall knowledge levels.

Finally, in the “Estimation of relevant clinical populations” section, participants indicated the types of patients they see in their practice and provided estimates of both how many live in their catchment area and the total population of that area. Participants read four clinical vignettes depicting potential BCI applications: one based on a recent example of invasive BCI use from the literature [4] and three based on clinical experiences of the primary investigator’s lab. Based on knowledge acquired from the survey and vignettes, participants rated clinical utility and impact on quality of life of various potential BCI applications adapted from a previous BCI survey [22].

### Prevalence estimation

To estimate the number of patients who might benefit from BCI, we used physicians’ estimates of their total catchment population and the number of patients in their catchment area with the following conditions: quadriplegic CP with preserved cognition, severe hemiplegic CP, hemiplegia from adult stroke, SCI (high cervical or thoracic injury), ALS or similar (loss of all motor control), spinal muscular atrophy (SMA) or severe muscular dystrophy (MD) or similar and brainstem stroke (locked-in syndrome/quadriplegia). We specified that “preserved cognition” refers to standard academic grade 1 level or higher, meaning patients can understand and follow simple instructions and pay attention to visual or auditory cues. The estimate for each condition was divided by the physician’s catchment area to derive prevalence estimates. We removed extreme outliers (3 times the interquartile range), then created a Canadian average and median prevalence for each condition, assuming the prevalence of these conditions is relatively homogenous across the Canadian population, as described previously [1]. We then multiplied the Canadian prevalence by 36,700,000, rounded from Statistics Canada’s population estimate of 36,708,083 from July 1st, 2017 [23]. The resulting average and median estimates of Canadians with each of these conditions were then added to produce an average and median estimate of all Canadians with the aforementioned conditions.

### Analysis

As per our consent process, incomplete surveys were excluded from analysis. Descriptive statistics were used to describe the following: participant demographics, participant responses to baseline BCI knowledge questions; physician subspecialty distributions; physicians’ patient demographics; and physicians’ rating of BCI applications and utility. A chi-square test was used to compare proportions of respondents across specialties. The Shapiro-Wilks test was used to check for normality and non-parametric tests were used when normality could not be assumed. A Kruskal-Wallis test was used to compare knowledge scores between all three specialties and a Mann-Whitney U test was used to compare BCI knowledge scores between adult and pediatric subspecialists in neurology and physiatry. An independent samples t-test was used to compare BCI knowledge scores between BCI-related and non-BCI-related subspecialties overall and within adult and pediatric neurology as well as between adult and pediatric specialists only in BCI-related subspecialties. One-way ANOVA was used to compare mean BCI knowledge scores across years of experience. Significance was considered at a level of *p* < 0.05. Statistical analysis was performed using IBM SPSS Statistics Version 24.

## Results

### Population

A total of 1713 eligible Canadian physicians were identified (Table 2). Valid emails were available for 922 physicians. Of these, 141 physicians responded, of which four were excluded (three incomplete responses and one not practicing in Canada), resulting in 137 completed submissions and an analyzable response rate of 14.9%. When comparing response rates by specialty, adult neurologists, pediatric neurologists, pediatricians and developmental pediatricians were all placed in the same group because physicians self-identified within these specialties. Responses were dependent on specialty (*p* < 0.05).

**Table 2:** Number of eligible physicians, valid emails collected and survey response rates by specialty and overall

	Eligible	Valid Emails	Number of Complete Responses (% of the study sample)	Analyzable Response Rate (%)
Adult Neurologists	1171*	664*	68 (49.6%)	16.1*
Pediatric Neurologists			39 (28.5%)	
Physiatrists	537	253	23 (16.8%)	9.1
Developmental Pediatricians (DP) and Pediatricians (P)	5 (4 DP, 1 P)	5 (4DP, 1 P)	7 (4 DP, 3 P) (5.1%)	100
Total	1713	922	137	14.9

Legend: *Note that adult and pediatric neurologists are grouped together in the “Eligible,” “Valid Emails” and “Analyzable Response Rate” sections because neurologists were able to self-identify as adult or pediatric specialists.

Table 3 summarizes physician participant demographics in terms of age, experience, clinician type and geographic distribution. The largest group of respondents, in terms of experience, had been in practice for 0 to 10 years (48.2%) and, in terms of practice type, were academic clinicians in either education or research (43.8%). All respondents were involved in clinical work of some kind. Hereafter, pediatricians and developmental pediatricians are analyzed as pediatric neurologists, as described in the Methods.

**Table 3:** Participant demographics

			A. Neuro	P. Neuro	Physiatry	Dev. Ped.	Pediatrics	Total
Demographics	n		68	39	23	4	3	137
	Gender (%F)		41%	59%	34%	75%	66%	46%
	Age	31 to 40	20	14	10	2	0	46
		41 to 50	25	12	6	1	0	44
		51 to 60	10	4	4	1	2	21
		61 to 70	9	8	2	0	1	20
		Greater than 70	4	1	1	0	0	6
	Years in practice	0 to 5	15	10	4	1	0	30
		6 to 10	20	9	6	1	0	36
		11 to 15	8	5	4	1	0	18
		16 to 20	6	3	1	0	0	10
		Greater than 20	19	12	8	1	3	43
	Clinician Type	Academic clinical researcher	14	5	1	1	0	21
		Academic clinician - research	14	12	4	0	0	30
		Academic clinician – education	14	8	5	2	1	30
		Academic clinician – administration	6	4	6	1	0	17
		Academic clinician	13	4	5	0	1	23
		Community clinician	6	3	2	0	1	12
		Academic researcher	0	0	0	0	0	0
		Other	1	3	0	0	0	4
Geography	Province	Alberta	21	9	9	2	0	41
		British Columbia	7	2	3	0	0	12
		Manitoba	3	4	2	1	1	11
		Newfoundland and Labrador	1	0	1	0	0	2
		Nova Scotia	1	2	0	0	0	3
		Ontario	20	16	6	1	1	44
		Quebec	15	6	0	0	1	22
		Saskatchewan	0	0	2	0	0	2

Legend: *A. Neuro* Adult Neurology, *P. Neuro* Pediatric Neurology, *Dev. Ped.* Developmental Pediatrics, *Ped.* Pediatrics. Categories with no respondents were not included in the table (e.g. Nunavut, Northwest Territories, Prince Edward Island … etc.)

### BCI awareness

**Fig. 1:**
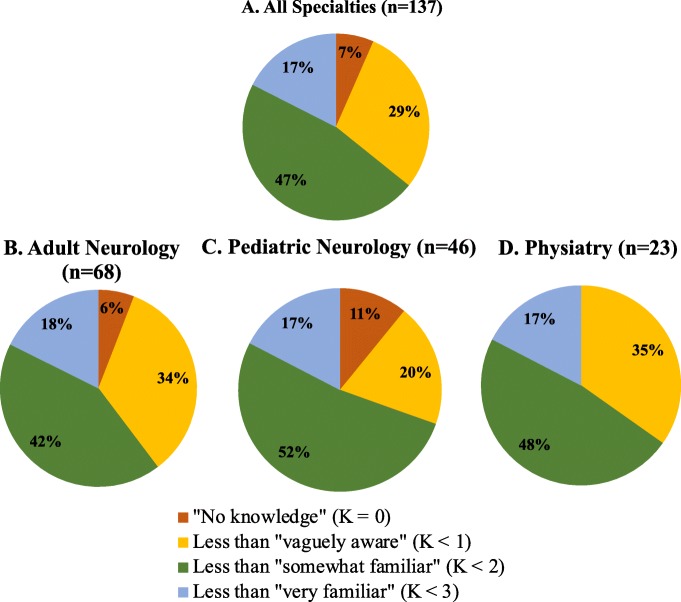
Distribution of BCI knowledge scores. Distribution of BCI knowledge scores (K) among all respondents (**a**) and by specialty (**b**, **c** and **d**).

Overall, clinician awareness of BCI technology was limited. Most physicians (83%) had BCI knowledge scores < 2, meaning their average level of knowledge was less than “somewhat familiar” (Fig. 1a). Only 17% of physicians had a score ≥ 2, meaning they felt “somewhat familiar” to “very familiar” on average (Fig. 1a). Figure 1b, c and d show the breakdown of knowledge scores by specialty.

**Fig. 2:**
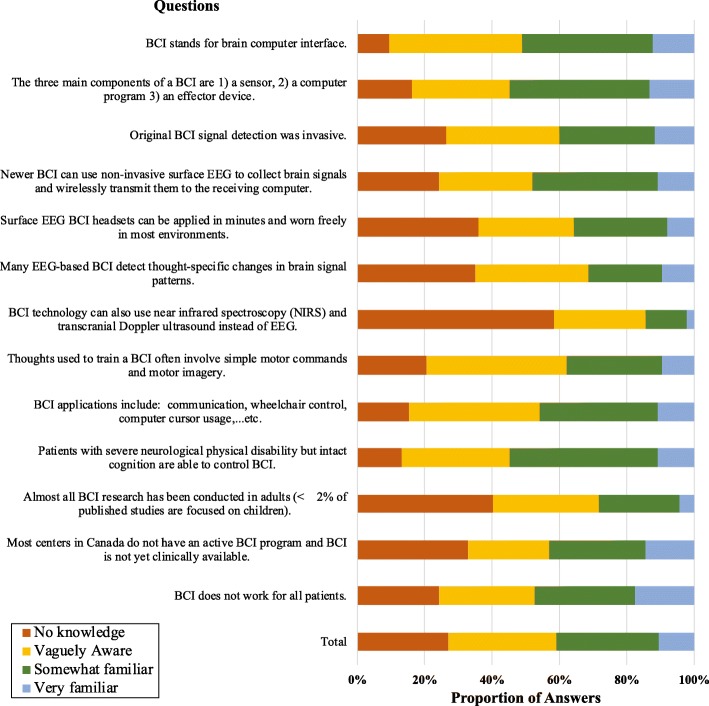
Physician responses to baseline BCI knowledge questions. See Additional file 1 for complete survey. The questions appear in the figure in the same order in which they appeared in the survey

Awareness varied by topic (Fig. 2). Physicians were least familiar with: 1) methods of recording brain signals, 2) the lack of pediatric BCI studies, and 3) surface EEG BCI headset application and wear. The statement physicians were most familiar with was that most centers in Canada do not have an active BCI program and that BCI is not yet clinically available.

**Fig. 3:**
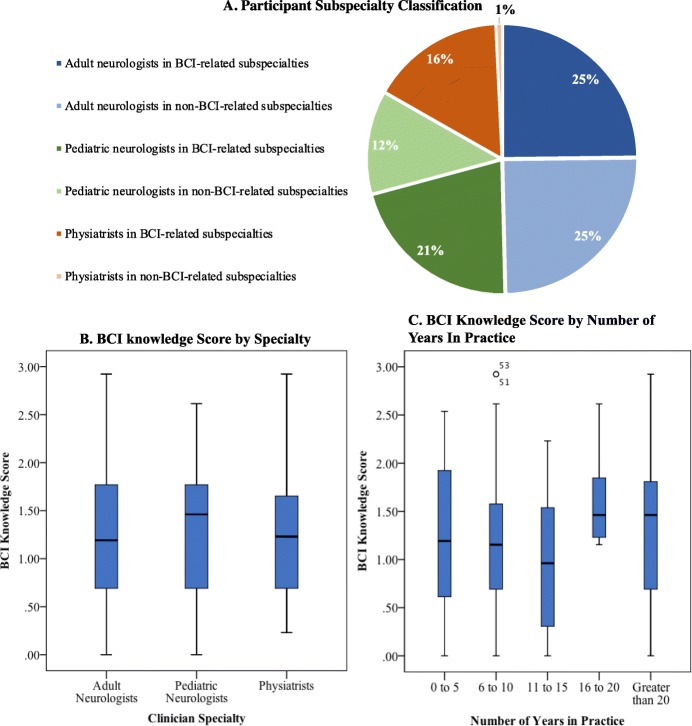
Participant Characteristics and BCI Knowledge. **a** Proportion of physicians practicing in subspecialties that are “BCI-related” and “non-BCI-related” separated by specialty. **b** Box and whisker plot indicating median, interquartile range and range of BCI knowledge scores across specialties (*n* = 137). Across specialties, there were wide ranges and comparable median scores. **c** Box and whisker plot indicating median, interquartile range and range of BCI Knowledge scores across different ranges of years in practice (*n* = 137). Across ranges of experience, there were wide knowledge score ranges and comparable median scores

The majority of physiatrists (62%) identified as practicing in at least one of the a *priori*-defined BCI-related subspecialties (Fig. 3a). Between specialties, there was no significant difference in baseline BCI knowledge scores (*p* = 0.808) (Fig. 3b). When physicians were dichotomized based on their self-identified “BCI-related” or “non-BCI-related” subspecialties, there was no difference in BCI knowledge across all specialties (*p* = 0.949) or either within adult neurology (*p* = 0.482) or pediatric neurology (*p* = 0.127). No difference was observed in BCI knowledge when comparing adult against pediatric neurologists in BCI-related subspecialties (*p* = 0.503). There was also no association between BCI knowledge and number of years in practice (*p* = 0.363) (Fig. 3c) or pediatric versus adult subspecialization across neurology and physiatry (*p* = 0.267).

**Fig. 4:**
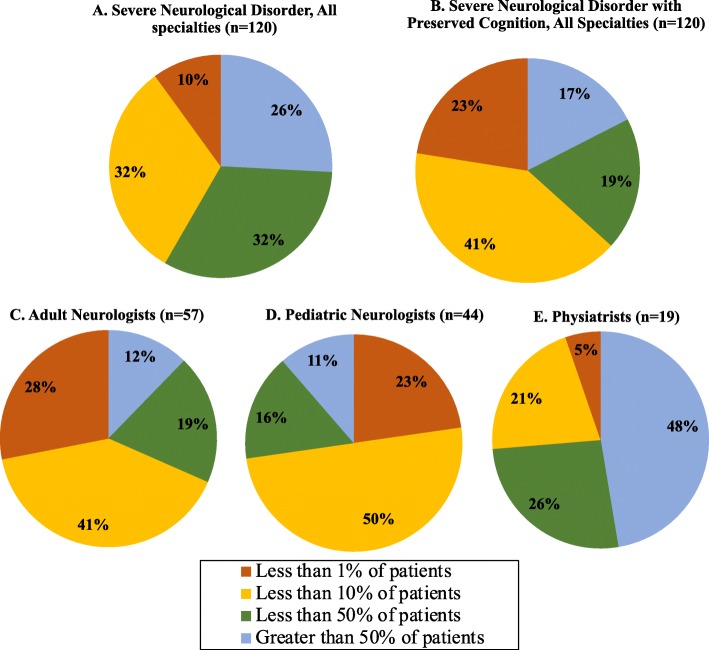
Proportions of patients in participants’ practices with SND overall and with preserved cognition. **a** Physicians (*n* = 120*) across all specialties reporting the proportion of patients in their practice with severe neurological disability (SND); **b** Physicians (*n* = 120*) across all specialties reporting the proportion of patients in their practice with severe neurological disability with preserved cognition (SNDwPC) (standard academic grade 1 level or higher); **c** Adult neurologists (*n* = 57*) reporting the proportion of patients in their practice with SNDwPC; **d** Pediatric neurologists (*n* = 44*) reporting the proportion of patients in their practice with SNDwPC; **e** Physiatrists (*n* = 19*) reporting the proportion of patients in their practice with SNDwPC. *Physicians who clearly misunderstood the question as judged by responses with inconsistent values were excluded

Thirty-one physicians (26%) reported that > 50% of their patients had a severe neurological disorder (SND) (Fig. 4a), while 21 (17%) reported that > 50% of their patients had a severe neurological disorder with preserved cognition (SNDwPC) (Fig. 4b). Most physicians (64%) reported that patients with SNDwPC represented < 10% of their practice (Fig. 4b). Notably, nearly half of physiatrists had practices where > 50% of patients had SNDwPC (Fig. 4e). Conversely, most pediatric neurologists (73%) had practices where < 10% of their patients had SNDwPC (Fig. 4d). Physicians’ BCI knowledge scores were not associated with the proportion of patients in their practice with SND alone (*p* = 0.266) or with SNDwPC (*p* = 0.173).

Only 4% of physicians (1 developmental pediatrician, 2 adult neurologists, 2 pediatric neurologists, 1 physiatrist) had patients currently using BCI, amounting to 21 patients total.

### Clinical applications

**Fig. 5:**
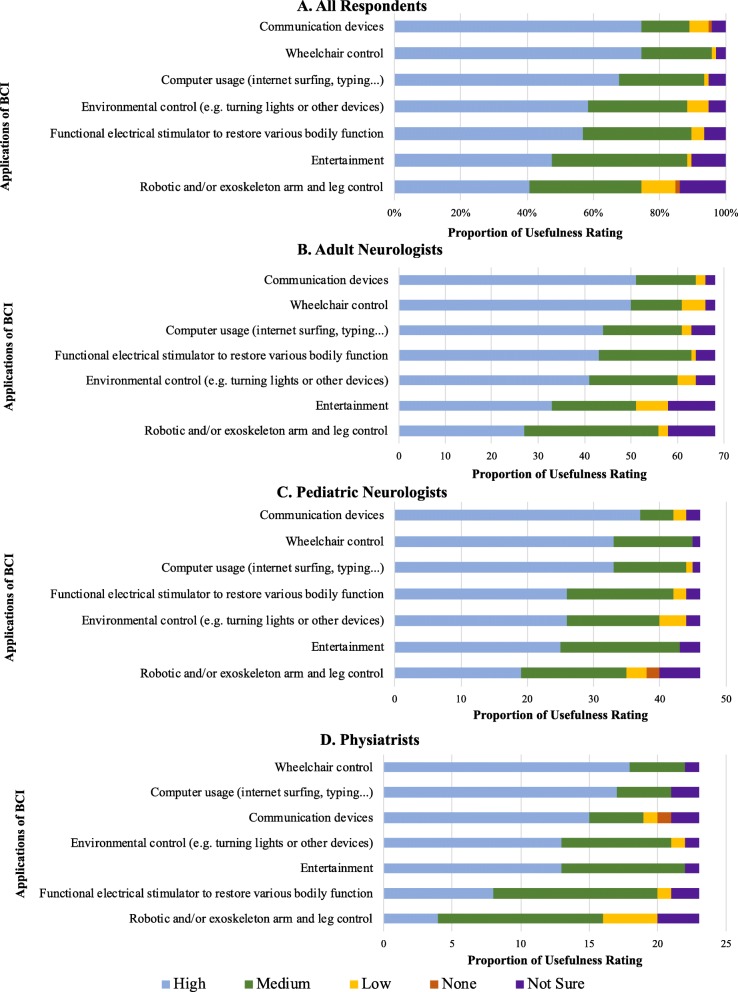
Physicians’ rating of potential usefulness of given applications of BCI overall and by specialty. Results are shown overall (**a**) and for each specialty (**b**: Adult Neurologists; **c**: Pediatric Neurologists; **d**: Physiatrists)

**Fig. 6:**
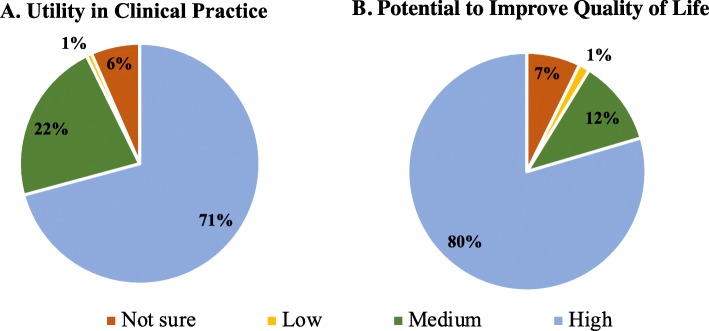
Utility of BCI in clinical practice and potential to improve quality of life. **a** Physicians’ rating of the utility of BCI in clinical practice. **b** Physicians’ rating of potential of BCI to improve quality of life

After reading the BCI knowledge statements and clinical vignettes, physicians most often rated communication devices as having the highest potential to improve quality of life, followed by wheelchair control and computer usage (Fig. 5a). These three applications were consistently rated the top three most useful by each specialty (Fig. 5b, c, d). However, the order was different among physiatrists, with wheelchair control first followed by computer usage then communication (Fig. 5d). 70% of participants rated BCI as having high utility in clinical practice (Fig. 6a), while 81% believe BCI has high potential to improve patient quality of life (Fig. 6b). Only 1% of participants thought BCI had low utility or potential to improve quality of life (Fig. 6a, b). 82.5% of physicians believed their patients would be open to adopting BCI.

### BCI-eligible population estimates

Based on physicians’ estimates of patient numbers with SNDwPC in their catchment area, there are likely somewhere between 13,000 and 32,000 Canadians who may benefit from BCI (see Table 4 for the disorders included in this estimate). Participants commented that patients with the following conditions may also benefit from BCI: sensory ganglionopathy, multiple sclerosis (advanced or quadriplegic), multiple system atrophy, limb malformation/amputation, severe traumatic or hypoxic brain injury, and severe Parkinson’s, Huntington’s, or other movement disorders.

**Table 4:** Average and median estimates of the Canadian prevalence for various neurological conditions

	Quadriplegic CP with preserved cognition (*n* = 55)	Severe hemiplegic CP (*n* = 48)	Hemiplegia resulting from adult stroke (*n* = 58)	Spinal cord injury (High cervical or thoracic injury) (*n* = 70)	ALS or similar (Loss of all motor control) (*n* = 51)	Spinal muscular atrophy or Severe muscular dystrophy or similar (*n* = 57)	Brainstem stroke (Locked-in syndrome/ quadriplegia) (*n* = 65)	Total
Average Prevalence (95% CI*)	5.4 per 100,000 (3.5–7.3)	10.4 per 100,000 (6.6–14.2)	50.9 per 100,000 (31.6–70.1)	12.9 per 100,000 (8.6–17.1)	4.1 per 100,000 (2.8–5.4)	3.1 per 100,000 (2.1–4.0)	0.7 per 100,000 (0.5–1.0)	-
Average Prevalence x CAD pop.** (× 10^3^) (95% CI*)	1.9 (1.3–2.7)	3.8 (2.4–5.2)	18.6 (11.6–25.7)	4.7 (3.1–6.3)	1.6 (1.0–2.0)	1.1 (0.8–1.5)	0.3 (0.2–0.4)	32 (20.4–43.8)
Median Prevalence (95% CI*)	2.5 per 100,000 (1–4)	5 per 100,000 (2–10)	20 per 100,000 (10–30)	4.5 per 100,000 (2–10)	2.5 per 100,000 (1.5–5)	1.5 per 100,000 (1–2.5)	0.3 per 100,000 (0.2–0.5)	-
Median Prevalence x CAD pop.** (×10^3^) (95% CI*)	0.9 (0.4–1.5)	1.8 (0.7–3.7)	7.3 (3.7–11.0)	1.7 (0.7–3.7)	9.2 (0.6–1.8)	0.6 (0.4–0.9)	0.1 (0.1–1.8)	13 (6.5–22.8)

Legend: *Data is not normally distributed but confidence intervals for mean calculated as if normality exists. Note that extreme outliers (3 times the interquartile range above the 3rd quartile) were excluded from the analysis. **Canadian population (CAD pop.) estimated at 36,700,000 based on Statistics Canada’s estimate on July 1, 2017 [23]

## Discussion

Our survey of 137 clinical specialists quantified multiple elements relevant to the advancement of clinical applications of BCI including clinician awareness, eligible clinical populations, and potential impact. Although the response rate was modest, the sample was diverse, including clinicians from eight out of ten provinces, with a variety of experience, practice types and subspecialisations. Most specialists encounter patients with severe neurological disability and cognitive capacity. Regardless of specialty or years of experience, BCI knowledge was poor and current patient BCI use rare. The vast majority of respondents endorsed BCI’s high potential to improve quality of life for severely disabled persons, with communication and mobility control rating the highest. Our estimates suggest that between 13,000 and 32,000 Canadians may benefit from BCI technologies.

Our results endorse the fundamental rationale for completing the study: the theoretical benefits of advancing BCI for severely disabled persons are high. While the literature generally agrees on BCI’s high potential to improve quality of life in patients with SND [2, 24–27], many studies also acknowledge that knowledge translation to clinical settings remains a major challenge. Our results demonstrating poor physician awareness of and familiarity with BCI across both pediatric and adult as well as “BCI-related” and “non-BCI-related” subspecialties are, therefore, not surprising. These findings reinforce the existing gap in knowledge translation toward experts working with populations most likely to benefit from BCI. Physician education on BCI has been endorsed in numerous studies as an untapped means of advancing BCI development and applications in clinical settings [25, 26, 28, 29].

In designing the survey’s “Baseline Knowledge” section, knowledge assessment questions were a series of statements that provided respondents with basic information about BCI, its potential clinical utility, and the current state of Canadian BCI programs. In this way, our survey both highlighted areas of BCI knowledge requiring better dissemination among clinicians as well as translated current knowledge from BCI literature. Health professionals should be primary targets to promote widespread use of research outcomes, including technologies [30], and researchers are a valuable source of new knowledge which may influence physician practice [30, 31]. Although a formal evaluation of knowledge transferred and retained was beyond this project’s scope, we hope our efforts have raised BCI awareness on a national scale.

Despite their limited awareness of BCI, respondents generally believed BCI technology has high utility in clinical practice and potential to improve quality of life, the major goal of BCI development [2, 26, 27]. Participants also believed their patients would be open to adopting BCI technology, a finding that may suggest enthusiasm among users as it becomes increasingly available. Despite our modest response rate, the strong endorsement of BCI’s high potential for clinical impact suggests many Canadian specialists may be interested in investing resources to advance BCI programs (though we failed to ask this question specifically).

While improving physician awareness to advance BCI applications is essential, patient engagement may be even more important. Literature regarding end-users’ functional priorities for BCI applications is inconsistent. Our physician participants rated communication devices, wheelchair control and computer usage as the three applications with the highest potential usefulness. These results are supported by a study of individuals with SCI which reported emergency communication, computer control and wheelchair control within the top four of 15 applications of BCI [32]. Further, a survey of happiness in patients with locked-in syndrome found unhappiness was most associated with limited mobility and poor recovery of speech production [33]. In contrast, priorities that our respondents ranked lower, such as extremity control and bowel/bladder function, have been prioritized in other studies of BCI-eligible populations [22, 34]. Hence, physician understanding of patients’ needs may not always align with patient priorities, demonstrating the importance of ensuring end-users of BCI technology are more engaged in future studies as well as BCI development programs and workshops at developer conferences and meetings.

Despite discrepancies between physician estimates and patient desires, however, clinicians have the potential to act as an essential link between researchers and technology developers and the patients who stand to benefit from their innovations [25]. Clinicians may be ideally positioned to collect and provide a platform that puts patient and family desires at the center of research while mitigating the ethical challenges of including such potentially vulnerable patients in studies. Emerging formal patient and family engagement methods, such as user-centred design, may further facilitate this process in future BCI research [35, 36].

The patient populations who stand to benefit from BCI are significant. We generated rough approximations that 13,000–32,000 Canadians are living with conditions that might benefit from BCI, including quadriplegic or severe hemiplegic cerebral palsy, severe hemiplegia from adult stroke, ALS or similar disorders, SMA/severe MD, or locked-in syndrome. Our techniques were limited by the nature of the study and we acknowledge that these numbers likely have only modest accuracy. However, we also believe this number may be an underestimate of the actual population for several reasons. First, participants identified many other patient populations not included in our total estimate who could potentially benefit from BCI, including patients with multiple sclerosis, multiple system atrophy, movement disorders, limb malformation, and severe traumatic or hypoxic brain injury. Further, emerging BCI applications, such as consciousness detection in intensive care settings or motor rehabilitation techniques [5], were also not included. These additional applications demonstrate the importance of raising awareness about BCI to help wider patient populations.

We also suspect our study may underestimate the underlying population because our numbers are consistently below prevalence estimates for neurological conditions reported by the Public Health Agency of Canada (PHAC) [37]. According to PHAC, per 100,000 Canadians, there may be 10 living with ALS, 130 with CP, 70 with muscular dystrophy, and 980 with stroke [37]. Other studies suggest that, per 100,000, the prevalence of CP may be 221 globally and the prevalence of SCI may be 4.23 in North America [1]. The discrepancy between our numbers and other studies’ may also be partly explained, however, by the fact that we asked respondents to consider only patients with specific qualities such as SNDwPC.

As expected, most physicians had relatively small proportions of patients with SNDwPC (< 10%). However, their disabilities are often most severe, resulting in greater need for technologies such as BCI [38]. Compared to patients with common neurological conditions, health services may be relatively scarce for rarer conditions [37]. Additionally, in patients with SNDwPC, failure to recognize preserved cognition may further compound barriers to accessing appropriate services. Under-estimating a person’s capacity based on external appearances of severe physical disability is a catastrophic mistake that might be directly reduced by improved BCI education and awareness amongst treating clinicians.

Despite limited BCI literature pertaining to children, pediatric specialists had similar knowledge levels to their adult counterparts, an encouraging result given the importance of advancing BCI use among pediatric populations. In Canada, more than 40% of children with neurological disability have limited educational opportunities and 15% are housebound [37]. Also, rehabilitation services accessible to children with newly diagnosed conditions tend to diminish over time [37]. Further, childhood neurological disabilities inherently carry a greater burden of disease throughout the lifespan for the child, caregivers and the community involved in their care. Though unproven, early intervention to introduce BCI applications during childhood may facilitate essential learning and social interactions at younger ages, yielding benefits across the lifespan such as increased adult capacity and higher lifelong function.

A number of important limitations are acknowledged. First, the possibility of selection responder bias is significant with a modest response rate, though different groups were equally represented. Physicians who answered the survey may have been more interested in BCI. Moreover, academic clinicians, who represented the largest group of participants, may be more likely than community physicians to see patients with complicated neurological conditions [19]. Our low response rate and resulting sample size also diminish generalizability of our results. Typically, survey response rates of 70% or above are required for external validity [39–41]. However, physician response rates tend to be lower with similar studies of neurologists and neurosurgeons responding at 32–47% [19, 42]. Our limited reminders, short data collection period, and English-only survey may have limited the response rate further. Our survey also only targeted physicians, excluding valuable insight from other relevant clinicians, including occupational, physical, and speech/communication therapists. These groups were excluded because we were unable to restrict our contact to the very small proportion of these specialists who work with relevant BCI-eligible populations. Hence, we did not want to compromise our results by acquiring opinions from a large proportion of professionals with little experience working with eligible populations. Questions regarding estimation of BCI clinical utility should be interpreted with consideration that many physicians completing our survey had little prior understanding of BCI. Finally, for questions that were not well-understood by participants, more extensive pre-testing and pilot testing may have reduced misinterpretation [43].

## Conclusions

In summary, we demonstrate that relevant clinicians in Canada have poor BCI awareness despite consensus that clinical utility is high. Ongoing efforts require better clinician, patient and family engagement in BCI development to optimize translation and improve the lives of people with severe disability.

## Supplementary information

**Additional file 1.** BCI survey sent out to participants.

## Data Availability

The datasets used and/or analysed during the current study are available from the corresponding author on reasonable request.

## Abbreviations

ALS: Amyotrophic lateral sclerosis
BCI: Brain computer interface(s)
CP: Cerebral palsy
EEG: Electroencephalography
MD: Muscular dystrophy
PHAC: Public Health Agency of Canada
RCPSC: Royal College of Physicians and Surgeons of Canada
SCI: Spinal cord injury
SMA: Spinal muscular atrophy
SND: Severe neurological disorder
SNDwPC: Severe neurological disorder with preserved cognition
