# Supplementary material for: Clinician awareness of brain computer interfaces: a Canadian national survey
Source: J Neuroeng Rehabil. 2020 Jan 6;17:2. doi: 10.1186/s12984-019-0624-7 (PMC6945584; doi:10.1186/s12984-019-0624-7)
Supplement: Supplementary file 1 — Additional file 1. BCI survey sent out to participants. [file 12984_2019_624_MOESM1_ESM.docx]

**Additional file 1: BCI survey sent out to participants**

**Part 1: Demographics**

Which of the following specialties are you currently licensed in and practicing in Canada?

**(dropdown, Required)**

| Pediatrics |
| --- |
| Pediatric Neurology |
| Neurology |
| Physical Medicine and Rehabilitation |
| Developmental Pediatrics |
| Not currently practicing in Canada |

**(Survey ends if participant selects “Not currently practicing in Canada”)**

**If participant selected “Neurology”:** Please select the area(s) in which you subspecialize: (Please check all that apply.)

**(checkbox, Required)**

| Alzheimer's disease |
| --- |
| Amyotrophic lateral sclerosis (ALS) |
| Acquired Brain Injury/Traumatic Brain Injury |
| Behavioural neurology |
| Brain tumour |
| Cerebral Palsy |
| Critical care |
| Epilepsy |
| Headache/Migraine |
| Movement disorders |
| Multiple sclerosis |
| Neuro-ophthalmology |
| Neuromuscular disorders |
| Neuro-oncology |
| Pain/Palliative |
| Sleep disorders |
| Spinal cord injury |
| Stroke |

**If participant selected “Pediatric Neurology”:** Please select the area(s) in which you subspecialize: (Please check all that apply.)

**(checkbox, Required)**

| Acquired Brain Injury/Traumatic Brain Injury |
| --- |
| Behavioural neurology |
| Brain tumour |
| Cerebral palsy |
| Critical care/Emergency neurology |
| Epilepsy |
| Headache/Migraine |
| Movement disorders |
| Multiple sclerosis |
| Neuro-ophthalmology |
| Neuromuscular disorders |
| Neuro-oncology |
| Pain/Palliative |
| Sleep disorders |
| Spinal cord injury |
| Stroke and perinatal stroke |

**If participant selected “Physical Medicine and Rehabilitation”:** Please select the area(s) in which you subspecialize: (Please check all that apply.)

**(checkbox, Required)**

| Acquired brain injury/Traumatic brain injury |
| --- |
| Electrodiagnostic medicine |
| Geriatric rehabilitation |
| Musculoskeletal medicine |
| Neuromuscular disorders |
| Paediatric rehabilitation |
| Pain management |
| Prosthetics and orthotics |
| Pulmonary, cardiac and cancer rehabilitation |
| Rheumatology |
| Spasticity Management |
| Spinal cord injury |
| Stroke |

Which of the following best describes your current position?

**(dropdown, Required)**

| Academic clinical researcher (Clinician scientist, majority of time in research) |
| --- |
| Academic clinician - research (Majority of time mixes clinical and research) |
| Academic clinician - education (Majority of time mixes clinical and education) |
| Academic clinician - administration (Majority of time mixes clinical and administration) |
| Academic clinician (Almost entirely clinical work in an academic setting) |
| Community clinician (Almost entirely clinical work in a non-academic setting) |
| Academic researcher (All research with no patient contact) |
| Other |

Years in practice:

**(dropdown, Required)**

| 0-5 |
| --- |
| 6-10 |
| 11-15 |
| 16-20 |
| >20 |

Age

**(dropdown, Required)**

| 20-30 |
| --- |
| 31-40 |
| 41-50 |
| 51-60 |
| 61-70 |
| >70 |

Gender:

**(radio, Required)**

| Male |
| --- |
| Female |
| Other |

Please indicate the province in which you practice:

**(dropdown, Required)**

| Alberta |
| --- |
| British Columbia |
| Manitoba |
| New Brunswick |
| Newfoundland and Labrador |
| Northwest Territories |
| Nova Scotia |
| Nunavut |
| Ontario |
| Prince Edward Island |
| Quebec |
| Saskatchewan |
| Yukon |

**If participant selected “Alberta”:** Please indicate the city in Alberta in which you practice or select other:

**(dropdown, Required)**

| Calgary |
| --- |
| Edmonton |
| Other |

**If participant selected “British Columbia”:** Please indicate the city in British Columbia in which you practice or select othe**r:**

**(dropdown, Required)**

| Abbotsford |
| --- |
| Burnaby |
| Coquitlam |
| Delta |
| Kelowna |
| Langley, District Municipality |
| Richmond |
| Saanich |
| Surrey |
| Vancouver |
| Other |

**If participant selected “Manitoba”:** Please indicate the city in Manitoba in which you practice or select other:

**(dropdown, Required)**

| Winnipeg |
| --- |
| Other |

**If participant selected “New Brunswick”:** Please indicate the city in New Brunswick in which you practice or select other:

**(dropdown, Required)**

| Moncton |
| --- |
| St. John's |
| Other |

**If participant selected “Newfoundland and Labrador”:** Please indicate the city in Newfoundland and Labrador in which you practice or select other:

**(dropdown, Required)**

| St. John's |
| --- |
| Other |

**If participant selected “Northwest Territories”:** Please indicate the city in Northwest Territories in which you practice or select other:

**(dropdown, Required)**

| Yellowknife |
| --- |
| Other |

**If participant selected “Nova Scotia”:** Please indicate the city in Nova Scotia in which you practice or select other:

**(dropdown, Required)**

| Halifax |
| --- |
| Other |

**If participant selected “Nunavut”:** Please indicate the city in Nunavut in which you practice or select other:

**(dropdown, Required)**

| Iqaluit |
| --- |
| Other |

**If participant selected “Ontario”:** Please indicate the city in Ontario in which you practice or select other:

**(dropdown, Required)**

| Ajax |
| --- |
| Barrie |
| Brampton |
| Burlington |
| Cambridge |
| Chatham-Kent |
| Guelph |
| Greater Sudbury |
| Hamilton |
| Kingston |
| Kitchener |
| London |
| Markham |
| Milton |
| Mississauga |
| Oakville |
| Oshawa |
| Ottawa |
| Richmond Hill |
| St. Catharines |
| Thunder Bay |
| Toronto |
| Vaughan |
| Waterloo |
| Whitby |
| Windsor |
| Other |

**If participant selected “Prince Edward Island”:** Please indicate the city in Prince Edward Island in which you practice or select other:

**(dropdown, Required)**

| Charlottetown |
| --- |
| Other |

**If participant selected “Quebec”:** Please indicate the city in Quebec in which you practice or select other:

**(dropdown, Required)**

| Gatineau |
| --- |
| Laval |
| Lévis |
| Longueuil |
| Montreal |
| Québec |
| Saguenay |
| Sherbrooke |
| Terrebonne |
| Trois-Rivières |
| Other |

**If participant selected “Saskatchewan”:** Please indicate the city in Saskatchewan in which you practice or select other:

**(dropdown, Required)**

| Regina |
| --- |
| Saskatoon |
| Other |

**If participant selected “Yukon”:** Please indicate the city in Yukon in which you practice or select other:

**(dropdown, Required)**

| Whitehorse |
| --- |
| Other |

If you selected "Other," please specify the city or town in which or out of which you practice.

**(text, Required)**

My estimated catchment population (all ages) is approximately:

**(dropdown, Required)**

| 50k |
| --- |
| 100k |
| 250k |
| 500k |
| 1M |
| 2M |
| 3M |
| 4M |
| 5M |
| 10M |
| More than 10M |

The approximate proportion of patients in my practice with severe neurological physical disability* is: 

*(e.g. quadriplegic cerebral palsy, severe hemiplegic cerebral palsy, hemiplegia resulting from adult stroke, spinal cord injury, ALS, spinal muscular atrophy, severe muscular dystrophy, brainstem stroke...etc.)

**(dropdown, Required)**

| < 1% |
| --- |
| < 10% |
| < 50% |
| >50% |

**Part 2: Baseline BCI Knowledge**

Please indicate your current personal level of knowledge regarding each of the following statements.

Note: These statements are also intended to serve as an introductory education for your ability to answer future sections.

| Statements | Possible Answers **(Required)** | | | |
| --- | --- | --- | --- | --- |
| BCI stands for brain computer interface. | No knowledge | Vaguely aware | Somewhat familiar | Very familiar |
| The three main components of a BCI are 1) a sensor to detect brain signals, 2) a computer program to interpret the signals obtained and detect specific patterns, and 3) an effector device to be controlled by these signals. | No knowledge | Vaguely aware | Somewhat familiar | Very familiar |
| Original BCI signal detection was invasive such as implanting small sensors in the motor cortex and connecting them via externalized wires to transmit information. | No knowledge | Vaguely aware | Somewhat familiar | Very familiar |
| Newer BCI can use non-invasive surface EEG to collect brain signals and wirelessly transmit them to the receiving computer. | No knowledge | Vaguely aware | Somewhat familiar | Very familiar |
| Surface EEG BCI headsets can be applied in minutes and worn freely in most environments. | No knowledge | Vaguely aware | Somewhat familiar | Very familiar |
| Many EEG-based BCI detect thought-specific changes in brain signal patterns. | No knowledge | Vaguely aware | Somewhat familiar | Very familiar |
| BCI technology can also use near infrared spectroscopy (NIRS) and transcranial Doppler ultrasound instead of EEG. | No knowledge | Vaguely aware | Somewhat familiar | Very familiar |
| Thoughts used to train a BCI often involve simple motor commands and motor imagery (e.g. thinking about squeezing left hand or right hand). | No knowledge | Vaguely aware | Somewhat familiar | Very familiar |
| BCI applications include: rehabilitation (e.g. BCI-controlled functional electrical stimulation of muscles to restore function), robotic arm or exoskeleton control, communication, wheelchair control, computer cursor usage, environmental control (e.g. turning lights or other devices on/off, opening doors, temperature control)...etc. | No knowledge | Vaguely aware | Somewhat familiar | Very familiar |
| Patients with severe neurological physical disability but intact cognition (e.g. patients with ALS, cervical spinal cord injury...etc) are able to control BCI. | No knowledge | Vaguely aware | Somewhat familiar | Very familiar |
| Almost all BCI research has been conducted in adults (< 2% of published studies are focused on children). | No knowledge | Vaguely aware | Somewhat familiar | Very familiar |
| Most centers in Canada do not have an active BCI program and BCI for the aforementioned applications is not yet clinically available. | No knowledge | Vaguely aware | Somewhat familiar | Very familiar |
| BCI does not work for all patients. | No knowledge | Vaguely aware | Somewhat familiar | Very familiar |

Please read the following educational clinical cases:

1. In 2016 in Minnesota, a man with quadriplegia secondary to cervical spinal cord injury was fitted with an implanted BCI and functional electrical stimulation (FES) system for his right upper extremity. Using his thoughts, he was able to reach, open his hand, grasp a cup of coins, lift it and pour accurately into another cup, then replace the cup on the table (Bouton et al., 2016).

2. In 2017 in Calgary, a 9 year-old boy with severe quadriplegic cerebral palsy was able to drive a remote-control car for the first time in his life using a commercially available surface EEG BCI after training for only 15 minutes.

3. In 2017 in Calgary, an 8 year-old boy with severe quadriplegic CP and normal intellect was able to play multiple, simple popular video games for the first time in his life using a surface EEG BCI after a single training session.

4. In 2017, a 39 year-old woman with locked-in syndrome following a brainstem stroke was able to control a computer screen cursor and video game using a simple EEG BCI with minimal training.

After reading the above clinical cases, how would you rate the utility of BCI in clinical practice (assuming it was available for patients to use)?

**(radio, Required)**

| Low |
| --- |
| Medium |
| High |
| Not Sure |

Have you previously used BCI technology to work with a patient?

**(radio, Required)**

| Yes |
| --- |
| No |

Please indicate which applications of BCI you have used to work with a patient. (Please check all that apply.)

**(checkbox, Required)**

| Communication |
| --- |
| Rehabilitation of motor function (e.g. BCI control of functional electrical stimulation of muscles to restore function) |
| Entertainment (using BCI to control computer games) |
| Environmental control (e.g. turning lights or other devices on/off, opening doors, temperature control) |
| Wheelchair control |
| Other |

If you selected "Other," please specify what you have used BCI for:

**(text, Required)**

**Part 3: Clinical Populations**

Which of the following types of patients have you encountered in your practice?

**(checkbox, Required)**

| Quadriplegic cerebral palsy (Non-ambulatory, minimal hand use but preserved cognition at standard academic grade 1 level or higher) |
| --- |
| Severe hemiplegic cerebral palsy (Paralyzed arm, BCI control of functional electrical stimulation to restore muscle function) |
| Hemiplegia resulting from adult stroke (Paralyzed arm, BCI control of functional electrical stimulation to restore muscle function) |
| Spinal cord injury (High cervical or thoracic injury) |
| ALS or similar (Loss of all motor control) |
| Spinal muscular atrophy/Severe muscular dystrophy or similar (Loss of all motor control) |
| Brainstem stroke (Locked-in syndrome/quadriplegia) |
| Other patient populations who could potentially benefit from BCI |
| None of my patients could potentially benefit from BCI |

Based on your practice and experience, please roughly estimate the number of patients in your catchment area with quadriplegic cerebral palsy and preserved cognition at standard academic grade 1 level or higher*: ______

*(i.e. patient can understand and follow simple instructions and pay attention to visual or auditory cues.)

**(text (number, Min: 0), Required)**

Based on your practice and experience, please roughly estimate the number of patients in your catchment area with severe hemiplegic cerebral palsy: ______

**(text (number, Min: 0), Required)**

Based on your practice and experience, please roughly estimate the number of patients in your catchment area with hemiplegia resulting from adult stroke: ______

**(text (number, Min: 0), Required)**

Based on your practice and experience, please roughly estimate the number of patients in your catchment area with spinal cord injury (high cervical or thoracic injury):

Based on your practice and experience, please roughly estimate the number of patients in your catchment area with ALS or similar (loss of all motor control): ______

**(text (number, Min: 0), Required)**

Based on your practice and experience, please roughly estimate the number of patients in your catchment area with spinal muscular atrophy/severe muscular dystrophy or similar (loss of all motor control): ______

**(text (number, Min: 0), Required)**

Based on your practice and experience, please roughly estimate the number of patients in your catchment area with brainstem stroke (locked-in syndrome/quadriplegia): ______

**(text (number, Min: 0), Required)**

If you selected "Other," please specify what other types of patients you are referring to and roughly estimate the number of patients in your catchment area with this condition: ***Condition (estimate)***

Drawing from your experience with patients and caregivers, please rate the following BCI actions in terms their potential impact on your patients' quality of life:

| **Application** | **Rating** | | | | |
| --- | --- | --- | --- | --- | --- |
| Computer usage (internet surfing, typing...) | None | Low | Medium | High | Not sure |
| Wheelchair control | None | Low | Medium | High | Not sure |
| Functional electrical stimulator to restore various bodily functions (e.g. hand movement, leg movement...) | None | Low | Medium | High | Not sure |
| Robotic and/or exoskeleton arm and leg control | None | Low | Medium | High | Not sure |
| Communication devices | None | Low | Medium | High | Not sure |
| Entertainment | None | Low | Medium | High | Not sure |
| Environmental control (e.g. turning lights or other devices on/off, opening doors, temperature control) | None | Low | Medium | High | Not sure |

Please specify any other applications of BCI that could improve your patients' quality of life (if applicable). *If none, please leave blank.*

Do you have patients who are already using BCI technology?

**(Dropdown, Required)**

| Yes |
| --- |
| Not that I know of. |

If you selected "Yes," please estimate how many of your patients are already using BCI: **(text, Required)**

Potential for BCI to improve quality of life:

**(radio, Required)**

| None |
| --- |
| Low |
| Medium |
| High |
| Not sure |

Do you think your patients would be open to adopting BCI technology?

**(radio, Required)**

| Yes |
| --- |
| No |
| I do not know |

|  |  |  |
| --- | --- | --- |
|  |  |  |
